# Supplementary material for: An analysis of farmers’ experiences with deterrence methods and investment in mitigation of agricultural crop damage caused by geese
Source: PLoS One. 2026 Feb 5;21(2):e0341807. doi: 10.1371/journal.pone.0341807 (PMC12875441; doi:10.1371/journal.pone.0341807)
Supplement: Attachment — Submitted filename: PLOS ONE response to reviewer comments.docx [file pone.0341807.s002.docx]

Response to reviewers

Academic editor:

1. Style requirements: We have checked and corrected author affiliations as well as the style of the body text.
2. Participant consent: We have provided more detailed information in the Methods section and hope that this is what is required.
3. Funding Statement: We do not have any changes to the original statement:

‘The project was primarily funded by a grant from the Danish Agency for Green Transition and Aquatic Environment to Jesper Madsen (www.sgav.dk)(Jagttegnsmidlerne 2023; ref. 120362), with support from a grant by the 15 June Foundation to Jesper Madsen ([www.15junifonden.dk](http://www.15junifonden.dk)) (project Samforvaltning i praksis – forsøg med bramgæs i Guldborgsund Kommune; ref. 2022-022).’

We can confirm that "The funders had no role in study design, data collection and analysis, decision to publish, or preparation of the manuscript."

4. Data availability: We have uploaded the datasets (in Danish) used for analysis to Zenodo, and it has been given the DOI 10.5281/zenodo.17936702.
5. Reviewer suggestion of additional reference: There was no suggestions made.
6. Reference list: the list has been checked.

Reviewer #1:

Line 352: "go out into field" not in table. I assume this should read: "walking person". Likewise, Figures 2 & 3 have listed "Walking man". This should be consistent with the other references to this hazing method.

**Response:** Changed to ‘walking person’ throughout and we have corrected and standardized the spelling of other hazing methods named slightly different in figures/texts/tables.

Figure 2/Line 373-375: Claims significance but does not state p-value in text. Horizontal lines indicating significance in the figure are distracting. Consider using error bars or indicate significance through: A, B, BC, C above the bar graphs.
**Response:** We have deleted horizontal bars and replaced them with letters above the bars to indicate which are statistically different (explained in the figure text), which has made the readability easier.

Overall, throughout text, be sure to use past-tense and fix grammatical errors, especially the use of ", and" at the end of sentences.
**Response:** has been checked and corrected. We present Methods in past-tense, and Results, Discussion and Conclusions in present-tense.

Line 394/Table 3/Figure 3: Throughout the publication, choose your desired p-value and stick with it. In the text/table 3, it appears that the p-value is referring to <0.001 suggesting. Then in figure 3, it appears that the error bars are set at maybe p-value of 0.05 (scare shots/walking man/driving car/atv all show similar significance, while in the table 3 they depict differences) . . . I think 0.05 is perfectly fine for these data but be consistent throughout.

**Response:** Please notice that we are dealing with categorical values for areas and times, and we have therefore shown median values and maximum/minimum values in Figure 1 and Figure 3 (not error bars), whereas in the pairwise comparisons in Table 2 and Table 3, we show the significance of posthoc pairwise tests. We see no reason to change this. We have consistently changed the p-values to the third significant decimal.

Table 4: Needs more explanation in the text and table (and format DF/p-value/F to be consistent at least). After reading numerous times, I am still not sure what table 4 is showing me. Season number vs hectare numbers that are of little value to the reader.

Table 5: Similarly, needs more explanation in text and formatting is too bad to interpret.
**Response:** We agree that the Table is not easy to read. Therefore, we have decided to delete Table 5 and only refer to the basic statistics in the text. We would like to retain Figure 5, however (see next response).

Figure 5: Needs more explanation in text and needs a more self-explanatory figure overall. (This data is covered exceptionally well in the discussion. Potentially remove the figure altogether and leave those data review for the text.)

**Response:** We prefer to keep the figure because this is the only visualization of the economic variables and their variation. We have tried to make the text more readable.

Table 6: Consider moving the "Hunters come ... " portion of the first column text to the top row and change the text to "Hunters available"

**Response:** thanks for the suggestion. We changed the table accordingly.

I was very impressed with the Discussion. The authors have a strong understanding of the issue. Likewise, I liked figures 1, 3, 4 and tables 2 and 3. Those were great visuals. Overall, well done!

**Response:** thanks!
